# More Lies Lead to More Memory Impairments in Daily Life

**DOI:** 10.3389/fpsyg.2022.822788

**Published:** 2022-02-22

**Authors:** Yan Li, Zhiwei Liu, Xiping Liu

**Affiliations:** ^1^Faculty of Psychology, Tianjin Normal University, Tianjin, China; ^2^School of Education and Psychology, Sichuan University of Science & Engineering, Zigong, China

**Keywords:** lying, cognitive resource, memory, memory-undermining effects, daily life

## Abstract

Previous studies have demonstrated that lying can undermine memory and that its memory-undermining effects could be modulated by the cognitive resources required to tell lies. We extended the investigation of the memory-undermining effect by using a daily life setting in which participants were highly involved in a mock shopping task. Participants were randomly assigned to truth-telling, denying or mixed lying conditions. After finishing the shopping task, participants were told that two people wanted to know about their shopping lists and would ask them some questions in an interview. During the interview, participants were asked whether each of ten items were on the shopping list, five of which were randomly selected from the shopping list, while the other five were not sold in the store. In answering the interview questions, the truth-telling group was asked to respond honestly, the denying group was asked to give denial responses, and the mixed lying group was asked to respond deceptively. Thus, the denying group told five lies and the mixed lying group told ten lies in the interview. The item memory test, source memory test and destination memory test were given in an orderly manner 48 h after the interview. We found that the mixed lying group, rather than the denying group, forgot the lies they told in the interview and mistakenly believed they had lied about something that they had not lied about. Moreover, the mixed lying group retained fewer memories about the person they responded to than the honest group. In addition, participants in the mixed lying group had more non-believed memories than those in the truth-telling group in both item and source memory tests. We conclude that more lies could result in more memory disruptions in daily life.

## Introduction

Lying, or deception, has been defined as a deliberate behavior that aims to give false information and induce false beliefs (Hyman, 1989; Vrij, 2004). It has been suggested that lying occurs every day (DePaulo et al., 1996; Riesthuis et al., 2021) and is essential for social interactions in daily life (Abe, 2011). Lying also happens in some special situations. Violent offenders often claim memory loss in an attempt to avoid legal responsibility or obstruct police investigations (Cima et al., 2002; Jelicic, 2018). Equally, some other offenders may come up with a fabricated version of their crime (Riesthuis et al., 2020). Moreover, victims of sexual abuse sometimes deny that the criminal act took place (Otgaar et al., 2016a). However, lying has consequences: liars’ memories may be affected by their lies (Pickel, 2004).

Extensive research has found that deception can undermine memory and has examined the effects of deception on memory as a function of the types of deception used. Generally, there are three types of deceptive strategies: false denial (denying events or details of events that happened), feigning amnesia (claiming to lose memories of events or details of events that one truthfully remembers), and fabrication (fabricating events or details of events that did not happen) (Otgaar and Baker, 2018). In the first study of false denial, Vieira and Lane (2013), using a within-subject design, found false denial to cause poor memory of the studied items compared to telling the truth. Studies using a between-subject design have also found that participants engaged in false denial make more omission errors than those who are truthful (Otgaar et al., 2016a, 2018, 2020; Romeo et al., 2019a; Battista et al., 2021a). Interestingly, previous studies have reported that false denial also results in worse performance on source memory tests relative to honest responses, suggesting that participants who deceive lose more memories about what they lied about (Otgaar et al., 2014a, 2016b). This pattern of results is defined as the denial-induced forgetting (DIF) effect (Otgaar et al., 2016a, 2018; Romeo et al., 2019a). It has also been suggested that falsely denying could create more non-believed memories than honesty (Otgaar et al., 2016a). Specifically, non-believed memories are memories that can be vividly recollected but no longer believed (Mazzoni et al., 2010; Otgaar et al., 2014b). In studies of feigning amnesia, more omission and commission errors in free and/or cued recall tests have been found in those who deceive than in those who are honest (Mangiulli et al., 2018a, b, 2019, 2020; Romeo et al., 2019b). Different from false denial and feigning amnesia, fabrication results in more false memories and memory distortions than telling the truth (Chrobak and Zaragoza, 2013; Otgaar et al., 2014a; Polage, 2019; Battista et al., 2020, 2021b). Therefore, the effects of deception on memory depend on the types of deception used.

Why do different types of deception result in different memory-disruption effects? Otgaar and Baker (2018) proposed a memory and deception (MAD) framework with which they argued that deception consumes cognitive resources and that different types of deception differ in cognitive resource consumption. False denial requires a few cognitive resources, feigning amnesia requires more resources than false denial, and fabrication demands more cognitive resources than the other two types of deception. Cognitive resource consumption leads to cognitive load. The more cognitive resources are consumed, the greater the cognitive load produced becomes. Therefore, the three types of deception lead to different cognitive loads and then cause different memory disruptions. In short, the key cause of memory impairments is cognitive resource consumption, and different memory outcomes result from different degrees of cognitive load.

Two studies have directly examined the idea that cognitive resource consumption is central to the observed memory-undermining effects. In their study, Battista et al. (2021a) manipulated three experimental conditions (simple false denial, complex false denial and truth-telling) that differed in cognitive resource consumption. A video depicting an electrician who stole several objects was presented in their study. The participants were asked to pretend they were eyewitnesses and engaged in a simulated police interview. During the interview, participants in the truth-telling group were asked to respond honestly, those in the simple false denial group were asked to give denial answers to all questions, and those in the complex false denial group were required to respond honestly to some questions and give false denial answers to the remaining questions. Clearly, many more cognitive resources were needed in the complex false denial condition than in the simple false denial and truth-telling conditions. The authors found the simple denial condition to lead to more memory impairment for the interview, while complex false denial incurred more memory disruption regarding the event. Therefore, their study demonstrates that a greater consumption of cognitive resources results in greater memory disruption.

Another study focused on the role of executive function (EF) resources in the effects of deception on memory. Battista et al. (2021c) hypothesized that the availability of EF resources could modulate the undermining effects of memory. In their study, two deceptive strategies, false denial and fabrication, were manipulated, and individuals’ EF resources were assessed. Memories of an event and interview were assessed. The authors found false denial to impair the participants’ memory of the interview and found fabrication to disrupt their memories of the event. Moreover, they demonstrated that individual differences in EF resources, especially in shifting resources, played an important role in memory-undermining effects. That is, individuals with more shifting resources could recall more correct details and had fewer omissions than those with fewer shifting resources regardless of which deceptive strategies they applied.

In the current study, we aimed to extend the investigation of memory-undermining effects depending on cognitive resource consumption. First, the two studies mentioned above used crime videos as materials. Crime is not a common occurrence for everyone and does not reflect the events of daily life. We wanted to determine whether the results of studies using crime videos could be generalized to everyday life. Second, in previous studies, participants were presented with crime videos, but they were not at the crime scene and had little involvement in the incident. It has been suggested that the degree of involvement could modulate the memory undermining effect (Li and Liu, 2021). Participants in high involvement conditions showed more memory disruptions and created more non-believed memories than those in low involvement conditions, and the DIF effect was found in the high but not in the low involvement conditions (Li and Liu, 2021). Moreover, the DIF effect was found to disappear when participants were highly involved and chose to lie when not instructed to (Li et al., 2022). In this study, we wanted to further test whether the memory-undermining effect would be modulated by cognitive resources for participants who are highly involved. Third, no previous studies on the subject of cognitive resource consumption have focused on destination memory, which is very important in helping liars keep their lies concealed. Destination memory, or target memory, refers to the memory of the person previously given information (Marsh and Hicks, 2002). For liars, destination memory is the memory of whom he or she has lied to. It has been suggested that destination memory could be disrupted when lying (Li and Liu, 2021; Li et al., 2022). Whether such disruption is modulated by deceptive strategies was also examined in the present study. Finally, it has been argued that memory and belief are distinct components (Scoboria et al., 2004, 2014) and can be differentially undermined by deception (Otgaar et al., 2014b, 2016a; Polage, 2017; Battista et al., 2020). Whether cognitive resource consumption modulates the effects of deception on memory and belief was also investigated in this study. We hope that the present research will contribute to a deeper understanding of the effects of deception on memory.

Following previous research (Li and Liu, 2021; Li et al., 2022), a daily life paradigm and a between-subject design were used in this study. The participants were asked to engage in a mock shopping task, buy some items in a store, and then complete a baseline memory test. Then, the participants were asked to answer questions about their shopping lists in an interview. For all of the questions, participants in the honest group were required to respond honestly, the denying group was asked to give denial responses, and the mixed lying group was asked to respond deceptively (i.e., denying and falsely reporting). To give a deceptive response to each question in the interview, participants in the mixed lying group may have needed to retrieve their shopping lists to determine whether they were being asked about the items on the shopping list, and then give an answer contrary to the truth. On the other hand, participants in the denying group may not have needed to retrieve their shopping list to determine whether the items they were being asked about were on their shopping lists and could instead simply give denial responses. Obviously, it is a more difficult task for the mixed lying group than for the denying group to give appropriate responses during the interview. Thus, more cognitive resources might be needed and more cognitive load might be produced for the mixed lying group than for the denying group. The final memory tests were given 48 h after the interview. Ratings on memory and belief were also recorded for each test. Based on previous studies (Battista et al., 2021a, c), we expected the mixed lying group to have more errors and create more non-believed memories on the final memory tests than the denying and truth-telling groups.

## Materials and Methods

### Participants

G*Power 3.1 (version 3.1.9.7) (Faul et al., 2007) was applied to determine the required sample size. Based on a previous work by Li et al. (2022), an *a priori* power analysis with an effect size of *f* = 0.36, a power of 0.80 and a significance level of α = 0.05 indicated that a sample of 78 participants was required. A total of 86 adults were recruited from a community in Tianjin. Four participants failed to complete the final memory tests due to personal reasons. Therefore, a final sample of 82 participants (*M*_age_ = 20.58, *SD* = 1.24, range: 18–23 years; 21 men) was available for data analyses. Participants took part in the present study voluntarily, and they were paid 35 yuan for their participation. The present study was approved by the ethical committees of Tianjin Normal University, and the participants provided informed consent in accordance with the principles of the Helsinki Declaration.

### Design and Procedure

A between-subjects design was used in this study (conditions: truth-telling, denying, and mixed lying). Dependent variables included error rates, memory and belief ratings, and number of non-believed memories on the memory tests. Participants were randomly assigned to one condition (Truth-Telling: *n* = 28; Denying: *n* = 26; Mixed Lying: *n* = 28). The study involved two sessions held 48 h apart.

#### Session 1

##### Shopping

We set up a small store in a room on the first floor of a building. Twenty kinds of products were for sale in the store: cookies, seaweed, bottled water, bread, gum, instant coffee, cola, strawberry pie, chocolate, instant noodles, soap, toothpaste, facial tissue, garbage bags, toothbrushes, hangers, N95 masks, towels, laundry detergent and cotton swabs.

Participants took part individually. They were asked to engage in a mock shopping task and buy ten items in the small store. No limit was set on their shopping time and lists. The participants were instructed to scan a QR code to complete a mock payment using their smartphones, although they did not pay any money. Then, a filler task (playing Tetris) lasting 5 min was given.

##### Baseline Memory Test

A baseline memory test followed the filler task. The participants were asked to freely recall and write down the items they bought. They were also asked to indicate their memory (Do you actually remember that you bought this item: 1 = *no memory at all*, 8 = *clear and complete memory*) and belief (How strong is your belief that you bought this item: 1 = *no belief*, 8 = *strong belief*) for each item. These scales were derived from the Autobiographical Belief and Memory Questionnaire (Scoboria et al., 2004, 2014). After finishing the baseline memory test, the participants completed another 5 min filler task (playing Tetris).

##### Interviews

The participants were told that two people on the second floor who did not know what they had bought would ask them questions about their shopping lists in an interview. The interviewers (both female) were strangers to the participants, and they were given the participants’ shopping lists when the participants were taking the baseline memory test.

Ten items were prepared for the interviews: five items not sold in the store (yogurt, a mirror, a cup, shampoo, and dried beef) and five randomly selected from the shopping lists. Using a fixed question structure (“Did you buy XXXX?”), the interviewers asked questions in an alternating order. One interviewer asked questions about the items on the shopping lists, and the other interviewer asked questions concerning the items not sold in the store.

Before the interviews, the participants were given instructions based on the experimental conditions. Participants in the truth-telling condition were asked to respond honestly to all of the questions. Participants in the denying condition were asked to give denial responses to all questions. Regardless of which items the interviewers asked about, participants in the denying condition were instructed to respond with “No, I did not buy XXXX.” In other words, the denying group was asked to falsely deny buying the items on their shopping lists and to state that the items were not on their shopping lists. Participants in the mixed lying condition were asked to give deceptive responses to all questions. Specifically, they were asked to respond “Yes, I bought XXXX” to questions about items not sold in the store (falsely reporting) and to respond “No, I did not buy XXXX” to questions about items on their shopping lists (denying).

#### Session 2

##### Final Memory Tests

Forty-eight hours after the interviews, the participants were asked to complete the final memory tests and respond honestly in the tests. In the final memory tests, an item memory test, a source memory test and a destination memory test were given in that order.

In the item memory test, the participants were asked to freely recall and write down the items they bought 2 days ago. Additionally, the participants were asked to indicate their memory (Do you actually remember that you bought this item: 1 = *no memory at all*, 8 = *clear and complete memory*) and belief (How strong is your belief that you bought this item: 1 = *no belief*, 8 = *strong belief*) for each item.

In the source memory test, twenty items were randomly presented to each participant: five items not sold in the store but asked about in the interviews, five items on the shopping list that were asked about in the interviews, five items not sold in the store and not asked about in the interviews (milk tea, a kettle, peanuts, a basin, and oatmeal), and five items on the shopping list and not asked about in the interviews. The participants were instructed to identify which items they had/had not been asked about in their interviews. Additionally, the participants were asked to indicate their memory (Do you actually remember that you were/were not asked about this item: 1 = *no memory at all*, 8 = *clear and complete memory*) and belief (How strong is your belief that you were/were not asked this item: 1 = *no belief*, 8 = *strong belief*) for each item.

In the destination memory test, the items that were asked about in the interviews and photos of the interviewers were presented to each participant. The participants were asked to identify who had asked them about particular items during the interviews from two photos of the interviewers. The participants were also asked to indicate the strength of their memory (Do you actually remember that this is the interviewer who asked you about this item: 1 = *no memory at all*, 8 = *clear and complete memory*) and belief (How strong is your belief that this is the interviewer who asked you about this item: 1 = *no belief*, 8 = *strong belief*) for each item.

For the baseline and item memory tests, we recorded the response accuracy based on the participants’ shopping lists. For the source and destination memory tests, we recorded the error rates based on the items asked about in the interview and the interviewer who asked about each item. Non-believed memories were also counted in the memory tests for each condition. Comparisons between the conditions were conducted to determine whether the differences in the dependent variables reached a significant level in the memory tests.

## Results

It has been argued that an analysis of variance (ANOVA) is not informative about the source of a main or interactive effect when experimental factors are of more than two levels (Schad et al., 2020). Therefore, the lme4 package (Bates et al., 2015) was applied to analyze all of the data in the R system (R Core Team, 2016). A linear mixed-effects model (LMM) was used to analyze memory and belief ratings for the correct responses, and a generalized linear mixed-effects model (GLMM) was used to analyze response accuracy, with participants and items measured as crossed random effects (Baayen et al., 2008). Following convention, *z* or *t* values greater than 1.96 are considered statistically significant.

**FIGURE 1:**
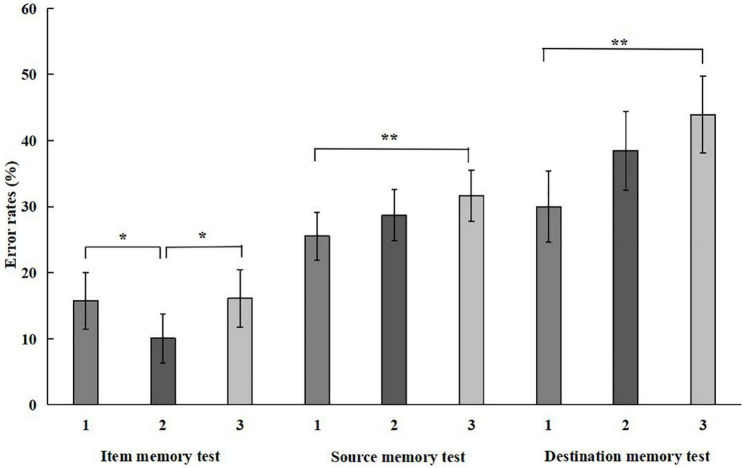
Mean error rates for item memory test, source memory test and destination memory test. 1, 2, and 3 represent Truth-Telling, Denying and Mixed lying conditions, respectively. Error bars show 95% confidence intervals. * < 0.05, ** < 0.01.

Descriptive and inferential statistics are shown in Tables 1, 2, and Figure 1.

**TABLE 1:** Mean error rates and belief and memory ratings with correct responses in memory tests for each condition.

		Truth-telling	Denying	Mixed lying
Baseline memory test	Error rate (%)	12.14 (1.96)	9.27 (1.81)	13.26 (2.03)
	Memory ratings	7.21 (0.08)	7.01 (0.09)	7.53 (0.06)
	Belief ratings	7.78 (0.05)	7.74 (0.06)	7.87 (0.03)
Item memory test	Error rate (%)	15.7 (2.18)	10.04 (1.87)	16.13 (2.21)
	Memory ratings	6.86 (0.09)	6.31 (0.11)	6.85 (0.11)
	Belief ratings	7.69 (0.06)	7.45 (0.08)	7.65 (0.08)
Source memory test	Error rate (%)	25.54 (1.84)	28.71 (1.99)	31.66 (1.97)
	Memory ratings	5.67 (0.11)	5.38 (0.11)	6.15 (0.1)
	Belief ratings	5.94 (0.11)	5.51 (0.12)	6.32 (0.24)
Item category 1	Error rate (%)	15.71 (3.08)	33.85 (4.17)	29.29 (3.86)
	Memory ratings	6.25 (0.19)	5.6 (0.22)	6.48 (0.17)
	Belief ratings	6.58 (0.18)	5.85 (0.23)	5.56 (0.17)
Item category 2	Error rate (%)	48.57 (4.24)	35.66 (4.23)	43.89 (4.22)
	Memory ratings	4.42 (0.27)	4.51 (0.23)	5.64 (0.23)
	Belief ratings	4.44 (0.27)	4.47 (0.24)	5.64 (0.24)
Item category 3	Error rate (%)	31.43 (3.94)	32.31 (4.12)	39.29 (4.14)
	Memory ratings	6.14 (0.19)	5.92 (0.21)	6.21 (0.21)
	Belief ratings	6.38 (0.21)	6.2 (0.21)	5.99 (0.22)
Item category 4	Error rate (%)	6.43 (2.08)	13.08 (2.97)	14.29 (2.97)
	Memory ratings	5.49 (0.21)	5.42 (0.19)	6.15 (0.22)
	Belief ratings	5.86 (0.19)	5.48 (0.21)	6.15 (0.2)
Destination memory test	Error rate (%)	30.01 (2.74)	38.46 (3.02)	43.93 (2.97)
	Memory ratings	5.19 (0.17)	4.19 (0.17)	5.03 (0.19)
	Belief ratings	5.25 (0.18)	4.62 (0.19)	4.92 (0.19)

*The standard error of the mean is shown in parentheses.*

*Item Categories 1, 2, 3, and 4 are items in the source memory test.*

*Item category 1, items that were on the shopping list and were asked about in the interview; Item Category 2, items that were on the shopping list and were not asked about in the interview; Item Category 3, items that were not sold in the store and were asked about in the interview; and Item Category 4, items that were not sold in the store and were not asked about in the interview.*

**TABLE 2:** Inferential statistics for each comparison and memory test.

Memory tests	Comparisons	Error rates				Memory ratings				Belief ratings			
		*b*	*SE*	*z*	*p*	*b*	*SE*	*t*	*p*	*b*	*SE*	*t*	*p*
Baseline memory test	1 vs 2	0.36	0.29	1.24	0.22	0.2	0.19	1.03	0.3	0.02	0.09	0.23	0.82
	1 vs 3	0.05	0.26	0.19	0.85	0.3	0.19	1.55	0.12	0.09	0.09	1.02	0.31
	2 vs 3	0.41	0.28	1.44	0.15	0.5	0.19	2.56	0.01	0.11	0.09	1.24	0.22
Item memory test	1 vs 2	0.61	0.3	2.01	0.04	0.57	0.3	1.87	0.06	0.24	0.16	1.49	0.14
	1 vs 3	0.05	0.28	0.17	0.87	0.03	0.3	0.11	0.92	0.05	0.16	0.34	0.74
	2 vs 3	0.56	0.3	1.87	0.06	0.54	0.3	1.77	0.08	0.18	0.16	1.16	0.25
Source memory test	1 vs 2	0.17	0.14	1.17	0.24	0.27	0.34	0.78	0.44	0.42	0.36	1.16	0.25
	1 vs 3	0.35	0.14	2.51	0.01	0.49	0.34	1.46	0.15	0.42	0.36	1.19	0.24
	2 vs 3	0.18	0.14	1.3	0.19	0.76	0.34	2.21	0.03	0.84	0.36	3.2	0.02
Item category 1	1 vs 2	1.19	0.39	3.06	0.002	0.54	0.41	1.32	0.19	0.62	0.39	1.61	0.11
	1 vs 3	0.98	0.39	2.53	0.01	0.31	0.4	0.78	0.43	0.04	0.38	0.1	0.92
	2 vs 3	0.21	0.36	0.6	0.55	0.87	0.42	2.08	0.04	0.66	0.4	1.66	0.1
Item category 2	1 vs 2	0.6	0.37	1.6	0.11	0.07	0.51	0.13	0.89	0.09	0.51	0.18	0.86
	1 vs 3	0.17	0.36	0.46	0.65	1.11	0.51	2.18	0.03	1.17	0.52	2.56	0.03
	2 vs 3	0.43	0.37	1.15	0.25	1.18	0.51	2.31	0.02	1.26	0.52	2.43	0.02
Item category 3	1 vs 2	0.04	0.26	0.16	0.88	0.18	0.34	0.52	0.61	0.16	0.36	0.44	0.66
	1 vs 3	0.38	0.26	1.48	0.14	0.18	0.34	0.53	0.6	0.26	0.37	0.71	0.48
	2 vs 3	0.33	0.26	1.3	0.19	0.36	0.35	1.02	0.31	0.1	0.37	0.27	0.79
Item category 4	1 vs 2	0.92	0.57	0.62	0.11	0.08	0.48	0.17	0.88	0.39	0.46	0.85	0.4
	1 vs 3	1.03	0.56	1.85	0.07	0.61	0.47	1.3	0.2	0.22	0.45	0.49	0.63
	2 vs 3	0.11	0.5	0.22	0.83	0.69	0.48	1.44	0.54	0.61	0.46	1.32	0.19
Destination memory test	1 vs 2	0.44	0.28	1.58	0.11	0.84	0.47	1.78	0.08	0.57	0.51	1.11	0.27
	1 vs 3	0.7	0.27	2.57	0.01	0.08	0.47	0.17	0.87	0.17	0.51	0.33	0.74
	2 vs 3	0.26	0.27	0.96	0.34	0.76	0.48	1.59	0.12	0.4	0.52	0.78	0.44

*1, truth-telling group; 2, denying group; 3, mixed lying group; vs, compared with.*

*Item Category 1, 2, 3, and 4 are the items in the source memory test.*

*Item Category 1, items that were on the shopping list and were asked about in the interview; Item Category 2, items that were on the shopping list and were not asked about in the interview; Item Category 3, items that were not sold in the store and were asked about in the interview; and Item Category 4, items that were not sold in the store and were not asked about in the interview.*

### Baseline Memory Test

As shown in Table 2, a significant difference was found between the denying group and the mixed lying group in memory ratings, suggesting that participants in the mixed lying group reported greater confidence in their memory of the shopping list than those in the denying group. No other significant differences were found in the baseline memory test results.

### Interviews

In the interviews, the participants were asked to respond according to the given instructions for each condition. Specifically, the truth-telling group was asked to tell the truth, the denying group was needed to give denial responses and the mixed lying group was required to respond deceptively to the questions (i.e., denying and falsely reporting). However, some false responses were given during the interview, and false responses were counted for each group (Truth-Telling group: 5 false responses; Denying group: 0 false responses; and Mixed lying group: 15 false responses). Chi-square analysis results suggest that more false responses were given by the participants in the mixed lying condition than those in the truth-telling (χ^2^ = 5.11, *p* = 0.02) and denying (χ^2^ = 14.33, *p* < 0.001) conditions. This pattern of results suggests that it was more difficult for the mixed lying group to give appropriate responses than the other two groups during the interview.

The items that were falsely responded to during the interview were excluded from the data analysis in the following memory tests, because the false responses for the truth-telling group were deceptive and those for the mixed lying group were honest.

### Item Memory Test

As shown in Table 2, the difference in error rates between the denying group and the truth-telling group reached a significant level, and a marginally significant difference was found between the mixed lying group and the denying group, suggesting that the denying group generated lower error rates than the other two groups. However, as shown in Table 2, two marginal significances were found in the comparisons of memory ratings, suggesting that the truth-telling and mixed lying groups had higher memory ratings than the denying group. No significant differences were found from other comparisons of the groups.

We also conducted a pre-post paired *t* test to compare the error rates of the baseline memory test and final item memory test for each group. No comparisons reached a significant level for the truth-telling group [*t*(27) = −1.91, *p* > 0.05], denying group [*t*(25) = −0.42, *p* > 0.05] or mixed lying group [*t*(27) = −1.25, *p* > 0.05]. These findings suggest that, compared with the baseline memory test, no groups had significant changes in error rates in the final item memory test.

There were ten items on the shopping list; five of them were asked about in the interview, and the other items were not. The items that were asked about may have been rehearsed during the interview and may have affected the final item memory test. Thus, we ran LMM analyses to determine whether the participants had better memories for the asked-about items than for the non-asked-about items. For all the participants, we found a significant difference in error rates between the asked-about items and the non-asked-about items in the final item memory test (*b* = 0.74, *SE* = 0.22, *z* = 3.42, *p* < 0.001), suggesting that the participants had better memories for the asked-about items than the non-asked-about items. For the participants in each group, we found that the memories for the asked-about items were better than those for the non-asked-about items in the truth-telling group (*b* = 1.19, *SE* = 0.41, *z* = 2.95, *p* < 0.01) and the mixed lying group (*b* = 0.89, *SE* = 0.37, *z* = 2.43, *p* < 0.05) but not in the denying group (*b* = 0.17, *SE* = 0.43, *z* = 0.41, *p* > 0.05).

### Source Memory Test

As shown in Table 2, a significant difference in error rates was found between the truth-telling and mixed lying groups, suggesting that participants in the mixed lying group forgot more items raised in the interviews than those in the truth-telling group. Moreover, the mixed lying group generated higher ratings on memory and belief than the denying group.

Separate analyses were also conducted based on the item categories, and the results are shown in Table 2. For items on the shopping list and asked about in the interviews, significant differences were found between the truthful and deceptive groups, suggesting that participants in both the denying and mixed lying groups had worse performance and were more forgetful in identifying this item category than those in the truth-telling group. The mixed lying and denying groups were asked to lie and falsely deny buying the items on their shopping lists during the interviews. Therefore, a DIF effect was found in the present study, both in the denying and mixed lying groups. The DIF effect indicates that participants in the deceptive groups forgot they had lied about the items on their shopping lists. Moreover, for the items not sold in the shop and not asked about in the interviews, a marginal difference in error rates was found to be statistically significant between the truth-telling and mixed lying groups, suggesting that the latter group had more errors in identifying items not asked about in the interviews.

Some significant differences were also found in the memory and belief ratings. For items on the shopping list raised in the interviews, participants in the mixed lying group had higher memory ratings than those in the denying group. For items on the shopping list but not raised in the interviews, the mixed lying group again had higher memory and belief ratings than the truth-telling and denying groups. No other significant differences were found for the source memory test.

### Destination Memory Test

A significant difference was found in error rates between the truth-telling and mixed lying groups, suggesting that participants in the mixed lying group forgot more of their targets. For participants in the mixed lying group, the response accuracy in identifying the target for each item raised in the interviews was approximately 56%, which is not much better than chance. No other comparisons reached a significant level.

### Non-believed Memories

As in previous studies (Clark et al., 2012; Otgaar et al., 2016a; Li and Liu, 2021; Li et al., 2022), non-believed memories were defined as items with memory ratings at least two scale points greater than belief ratings regardless of response correctness. For example, a participant reported a memory rating of 8, indicating a strong memory, and a belief rating of 6, indicating a moderate belief score for an event; this was classified as a non-believed memory.

The number of non-believed memories of each memory test was counted and is shown in Table 3. Chi-square analyses were conducted to determine whether deception created more non-believed memories than honest memories. Significant differences were found between the truth-telling and mixed lying groups for the item memory (χ^2^ = 4.57, *p* = 0.03) and source memory tests (χ^2^ = 9.55, *p* = 0.002), suggesting that lying created more non-believed memories in the memory tests. No other significant differences were found.

**TABLE 3:** The number of non-believed memories in the memory tests for each condition.

	Truth-telling	Denying	Mixed lying
Baseline memory test	7	3	5
Item memory test	1	2	7
Source memory test	20	31	44
Destination memory test	20	20	24

## Discussion

Previous studies have demonstrated that the undermining effects of memory could be more significant when liars employ a deceptive strategy that requires more cognitive resources (Battista et al., 2021a, c). The present study was carried out with the aim of verifying the effects of deceptive strategies on memory outcomes using a daily life paradigm. In line with our expectations, the mixed lying group had more errors and non-believed memories in several memory tests than the truth-telling group. On the other hand, denying did not result in more impairments in memory tests than truth-telling behavior. Therefore, consistent with previous studies (Battista et al., 2021a), this study supports the idea that lying but not simply denying could bring more memory disruptions.

Surprisingly, participants in the denying group presented lower error rates in the item memory test than members of the truth-telling and mixed lying groups. This finding shows that participants in the denying group had more correct memories of what they had bought 2 days ago than those in the other two groups. This finding is in line with a previous study (Battista et al., 2020, 2021a) but inconsistent with other studies (Vieira and Lane, 2013; Romeo et al., 2019a; Otgaar et al., 2020). A possible explanation for this discrepancy may be due to the retrieval-induced forgetting effect, which has been widely observed in previous studies (Racsmany and Keresztes, 2015; Pica et al., 2018; Abel and Bäuml, 2020). More specifically, to give an honest or deceptive response to each question during the interview, truth-telling and mixed lying groups might have needed to retrieve their memory to determine whether the asked-about item was on their shopping list or not and then to give a Yes or No answer. However, the participants in the denying group probably did not need to retrieve their memories but simply to give a denial response, regardless of whether the asked-about item was on their shopping list. It has been suggested that the retrieval-induced forgetting effect occurs when people are asked questions concerning eyewitness information because they need to retrieve their memories to answer the questions (Camp et al., 2012). The same situation may have occurred in the truth-telling and mixed lying groups in the present study, causing these groups to perform worse in the item memory test than those who may not have needed to retrieve their memories, such as the denying group. The evidence for this possibility is that the truth-telling and mixed lying groups, but not the denying group, were found to have better memories of the asked-about items than the non-asked-about items. The asked-about items may have been retrieved and rehearsed for the truth-telling and mixed lying groups; thus, their memories of the asked-about items were better than their memories of the items that were not asked about in the interview. Therefore, the findings observed in the final item memory test probably result from the retrieval-induced forgetting effect. More work, however, is needed to test this possibility. Another possible reason for the finding in the final item memory test is related to individual differences among groups. The denying group already committed fewer errors in the baseline memory test than the other groups (though the difference was not significant), and we found no significant differences when comparing the baseline memory test and the final item memory test for each group. These findings may suggest that memories of shopping lists were not forgotten for each group after the interview, and the significant differences found in the final item memory test might be due to the individual differences among groups, the participants were assigned to each group randomly though.

Moreover, for the item memory test, no significant differences in error rates were found between the truth-telling and mixed lying groups, which is consistent with some previous studies (Otgaar et al., 2016a, 2018; Battista et al., 2021a; Li and Liu, 2021; Li et al., 2022) but inconsistent with others (Vieira and Lane, 2013; Romeo et al., 2019a; Otgaar et al., 2020). This inconsistency may be due to the high degree of involvement employed in this study. The participants were asked to engage in a mock shopping task. They were thus highly involved and could obtain visual and tactile information during the shopping experience, and this information was essential to their item memories. Thus, no effects of lying on item memory were found in the item memory test. Another possible reason for this observation is related to the responses for the truth-telling and mixed lying groups. The truth-telling and mixed lying groups needed to recall their shopping lists and give honest or deceptive responses regarding the asked-about items during the interview. Thus, the asked-about items may have been retrieved and rehearsed during the interview for the truth-telling and mixed lying groups. Moreover, the non-asked-about items may have not been rehearsed for the two groups. Therefore, the differences in error rates did not reach significant level between the truth-telling and mixed lying groups, which may be due to their retrieving and rehearsing memory processes in the interview.

For the source memory test, significant differences in error rates were only found between the mixed lying and truth-telling groups. This pattern of results means that the participants in the mixed lying group, but not those in the denying group, forgot more items raised in the interviews than those in the truth-telling group. Moreover, we found that the mixed lying group presented higher belief and memory ratings for correct responses than the denying group. This finding means that the mixed lying group rated their belief and memory scores higher than the denying group. The denying group was asked to give denial responses to every interview question regardless of whether the items raised were on their shopping lists. The mixed lying group needed to falsely deny buying items on their shopping lists and falsely report buying those not on their shopping lists. This was a more difficult task, potentially requiring the mixed lying group to use more cognitive resources to provide responses in the interviews. It was necessary for the mixed lying group to determine whether the asked-about items were on their shopping lists and then construct deceptive lies for them. Therefore, the mixed lying group gave higher memory and belief ratings than the denying group for correct responses in the source memory test, although the two groups showed no differences in error rates.

Separate analyses of the source memory test based on item categories reveal interesting results. First, a DIF effect was found in the deceptive groups for items for which they gave false denial responses in the interviews. This finding is consistent with previous studies (Otgaar et al., 2014a, 2016a, 2018, 2020; Battista et al., 2021a; Li and Liu, 2021; Li et al., 2022) and suggests that liars forgot what they had falsely denied in the interviews. Partly inconsistent with the work by Battista et al. (2021a), the present study also found a DIF effect in the mixed lying group. This inconsistency may be attributable to the differences in the manipulated lying groups between the two studies. The mixed lying group in the previous work was asked to falsely deny some questions, which required the participants to remember the questions before the interview and give denial responses in the interviews (Battista et al., 2021a). However, the mixed lying group in the present study did not need to remember items before the interviews (because they did not know which items would be raised in the interviews) and was asked to give deceptive responses in the interviews. Remembering items before an interview may relieve the difference between the mixed lying and honest groups and cause the DIF effect to disappear. Moreover, the mixed lying group, but not the denying group, reported more errors than the truth-telling group on items not on their shopping lists and not asked about in the interviews. This finding suggests that participants in the mixed lying group mistakenly believed that they had lied about items not asked about in the interviews. In other words, participants in the mixed lying condition believed that they had lied about items that they had not lied about. Thus, participants in the mixed lying group not only forgot the lies they told but also had false memories about lies they had not told. This pattern of results is consistent with previous studies (Li and Liu, 2021; Li et al., 2022). There are several possible explanations for this result. One possible explanation might be related to the substantial number of lies that the mixed lying group told in a short period of time. This may have led these individuals to overestimate the number of lies they told and may have affected their memory of items not asked about in the interviews. Another possible explanation is that more cognitive resources were used by the mixed lying group during the interviews. Thus, it was difficult for these individuals to remember which lies they had told, leading to false memories of items they had not lied about. On the other hand, the denying group was only asked to give denial responses to interview questions, thus requiring fewer cognitive resources to complete the task and not resulting in more errors in identifying items not sold in the shop than for the honest group. Moreover, this result may also be caused by failed source monitoring for the mixed lying group. It has been argued that source misattributions or reality monitoring failures can lead to memory distortion (Johnson, 1997). Previous studies have demonstrated that source monitoring errors may be one of the reasons that lead to memory distortion when people lie (Pezdek et al., 2009; Chrobak and Zaragoza, 2013; Stolzenberg and Pezdek, 2013). In the present study, participants in the mixed lying group had more memory distortions than those in the truth-telling group in the source memory test, reflecting that the liars failed to identify the source of asked-about items in the interview. This result might be explained by the fact that the mixed lying group failed to monitor the source when they lied in the interview.

Another interesting finding was obtained from the destination memory test. Generally, liars need to remember their lies and whom they have lied to keep lies concealed and avoid inconsistency in daily life. However, participants in the mixed lying group, but not those in the denying group, almost completely forgot who their targets were and performed worse than those in the truth-telling group. This result is consistent with previous studies (Li and Liu, 2021; Li et al., 2022) and may be explained by the possibility that the mixed lying group needed more cognitive resources to lie, which created greater cognitive loads when lying. The participants were required to give deceptive responses to all interview questions. First, they needed to verify whether the item being asked about was on their shopping list; then, they suppressed the truth and gave a deceptive response. On the other hand, the truth-telling group did not need to suppress the truth and gave honest responses instead. Thus, more cognitive resources might have been needed and more cognitive load might have been created for the mixed lying group. The mixed lying group needed to direct more attention toward lying than toward whom they were lying to. Therefore, participants in the mixed lying group had forgotten more memories than those in the truth-telling group in the destination memory. On the other hand, the denying group gave denial responses and the honest group gave correct answers to the interview questions. Responding to the interview questions was simple for the honest and denying groups; thus, they might have had more cognitive resources available to pay attention to the interviewers and did not show any differences in the destination memory test results. Alternately, another possible explanation for the observations in the destination memory test is related to the coding of context. It has been demonstrated that memory processes can be impacted by context (Smith et al., 1978, 2018). There was a social context when participants answered the questions in the interview. There may have been some differences between the mixed lying and honest groups when the participants encoded the interview context, such as the questions and the interviewers. In other words, the groups might have differed in the coding of context during the interview, and the (dis)honest context may play a role in the findings obtained in the destination memory.

Lying also created more non-believed memories than truth-telling in the item and source memory tests. Participants in the mixed lying group identified more items that they were uncertain about than participants in the truth-telling group. However, no differences in non-believed memories were found between the denying and truth-telling groups. These results are consistent with previous studies (Otgaar et al., 2014b, 2016a; Polage, 2017; Battista et al., 2020; Li and Liu, 2021; Li et al., 2022) showing that deception may create more non-believed memories than honesty. Several factors may explain these observations. Using a daily life paradigm, participants in the present study were asked to engage in mock shopping in a store, and they were highly involved in the task. Participants in the denying group gave denial responses, and those in the mixed lying group were asked to respond deceptively to all questions. Fewer cognitive resources might have been needed by participants in the denying group, and more cognitive resources might have been needed for participants in the mixed lying group. A high degree of involvement and the need for fewer cognitive resources may have prevented the denying group from creating more non-believed memories than the truth-telling group. However, participants in the mixed lying group might have needed considerably more cognitive resources, which produced a greater cognitive load, resulting in the high degree of involvement playing a limited role in reducing non-believed memories for these individuals. Another possible reason for the finding observed in the destination memory may be related to the contextual coding in the interview. The participants in the mixed lying group may have applied a coding method, which may have differed from that used by the other two groups, to encode the interview context, such as the asked-about items and the interviewer who asked about a specific item when they gave dishonest answers during the interview.

This study contributes to a better understanding of the studied memory-undermining effect. First, we extend the findings of studies focused on viewing videos of criminal activity to daily life settings. Consistent with previous studies (Battista et al., 2021a, c), lying strategies that may require more cognitive resources could impair memory more than those that require fewer cognitive resources. Cognitive resource consumption might modulate the effects of deception on memory. Second, the participants in the mixed lying group were asked to falsely report buying items not on their shopping lists during the interviews. As shown in Table 2, such false reporting was not found to impair source memory in the present study (see the results for category 3), which is consistent with some previous studies (Li and Liu, 2021; Li et al., 2022) but inconsistent with others (Davis et al., 2018; Polage, 2019). This inconsistency might be due to the tasks used in various studies. A life events inventory was used in the work by Davis et al. (2018) and Polage (2019), and their participants were asked to determine whether events mentioned in the inventory occurred before they were 10 years of age. In the present study, the participants were required to determine whether the items presented in the source memory test were raised in the interview 2 days prior. Variations in the recentness of falsely reported events might have led to inconsistency between studies. It might be that false assent influences views of events occurring years ago but does not create impairment for recent events. More work, however, is needed to test this possibility. Third, a DIF effect was found in both the denying and mixed lying groups in the present study. The mixed lying group needed to respond deceptively to each interview question while the denying group gave denial responses regardless of whether the items raised were on their shopping lists. More cognitive resources might be needed for the participants in the mixed lying group than for those in the denying group in the interviews. However, we did not find the appearance of DIF to be modulated by deceptive strategies in the present study, which is not consistent with previous work (Battista et al., 2021a). As mentioned above, differences in the manipulation of lying might have caused such inconsistency. Finally, we found that the mixed lying group, but not the denying group, showed more errors in destination and more non-believed memories in the item and source memory tests than the honest group. These findings are novel and suggest that the consumption of cognitive resources might modulate the observed memory-undermining effects. Liars’ memories may be more disrupted when they tell more lies because they may need more cognitive resources to suppress the truth and construct lies, leaving fewer cognitive resources available to remember other details central to their lies, such as the people they have lied to. A more general explanation, as mentioned above, is that the mixed lying group may have had some differences in coding the interview context compared with the coding used by the other groups. The required dishonest responses in the interview may have led the mixed lying group to apply a contextual coding strategy, which may have differed from the coding employed by the other groups and caused the mixed lying group to experience more memory impairments for the interview.

Our observations have theoretical and practical implications. First, the mixed lying and denying groups mainly differed in required responses in the interview, and we found that the mixed lying group gave more false responses during the interview. Thus, cognitive resource consumption may have differed between the mixed lying and denying groups, and the mixed lying group may have required more cognitive resources and experienced more cognitive load than the denying group. In the present study, we found that the mixed lying group, but not the denying group, made more errors on source and destination memory tests and created more non-believed memories in the item and source memory tests than the honesty group. These findings support the MAD framework (Otgaar and Baker, 2018), which argues that deceptive strategies require more cognitive resources and may cause more memory disruptions. Second, consistent with previous studies (Otgaar et al., 2018, 2020; Romeo et al., 2019a, b; Battista et al., 2020, 2021a), we found that participants in the mixed lying group forgot what they had lied about in the interviews and mistakenly believed that they had lied about some items that they had not lied about. The mixed lying group also gave more false responses in the interviews and was more likely to forget whom they had responded to. Therefore, it is dangerous for us to tell many lies within a short period of time in our daily lives because we might forget the lies we have told and whom we have lied to, increasing the likelihood that our lies will be discovered.

In summary, we investigated the effects of lying on memory using a daily life paradigm in the present study. We found that the memory-undermining effect could be modulated by deceptive strategies that may differ in cognitive resource consumption during lying. Specifically, telling more lies may require more cognitive resources when lying, bring more disruptions in source and destination memory, and create more non-believed memories. A lying strategy that may consume fewer cognitive resources, such as denying, often results in a less memory-undermining effect. In daily life, the more lies we tell in a short time, the more memory impairments we may incur.

## Data Availability Statement

Data files are available online in the Figshare repository: https://figshare.com/s/ec0fc01e825481aa4e7c.

## Ethics Statement

The studies involving human participants were reviewed and approved by the Faculty of Psychology at Tianjin Normal University. The patients/participants provided their written informed consent to participate in this study.

## Author Contributions

YL designed the experiments and the stimuli and collected the data with help from ZL, YL wrote the manuscript with the help from ZL and with critical comments from XL. ZL analyzed the data. All authors contributed to the article and approved the submitted version.

## Conflict of Interest

The authors declare that the research was conducted in the absence of any commercial or financial relationships that could be construed as a potential conflict of interest.

## Publisher’s Note

All claims expressed in this article are solely those of the authors and do not necessarily represent those of their affiliated organizations, or those of the publisher, the editors and the reviewers. Any product that may be evaluated in this article, or claim that may be made by its manufacturer, is not guaranteed or endorsed by the publisher.
